# Editorial: New Perspectives on the Endocrinology of Physical Activity and Sport

**DOI:** 10.3389/fendo.2021.728756

**Published:** 2021-09-16

**Authors:** Kathryn E. Ackerman, Katia Collomp, Claudio E. Kater, Flavio Adsuara Cadegiani

**Affiliations:** ^1^Divisions of Sports Medicine and Endocrinology, Boston Children’s Hospital, Boston, MA, United States; ^2^Neuroendocrine Unit, Massachusetts General Hospital, Boston, MA, United States; ^3^Harvard Medical School, Boston MA, United States; ^4^CIAMS, Université d’Orléans, Orléans, France; ^5^Université Paris-Saclay CIAMS, Orsay, France; ^6^Département des Analyses, AFLD, Chatenay-Malabry, France; ^7^Endocrine Division, Department of Medicine, Escola Paulista de Medicina, Universidade Federal de São Paulo, São Paulo, Brazil; ^8^Applied Biology Inc, Irvine, CA, United States; ^9^Department of Endocrinology, Corpometria Institute, Brasilia, Brazil

**Keywords:** sports endocrinology, biomarkers, overtraining syndrome, relative energy deficiency in sport, athlete

This edition of Frontiers in Endocrinology includes a compilation of papers seeking to determine endocrine markers that could be useful to predict performance, monitor training load, and assess low energy availability (EA) risk. We recommend beginning with Kraemer et al., who provide an in-depth review of complex interactions among androgens, growth hormones (GHs), insulin-like growth factor 1 (IGF-1) and its superfamily, glucocorticoids, and various binding proteins, regulation, and signaling pathways in the setting of exercise and circadian influence. Improved understanding of these anabolic:catabolic mechanisms will help the reader appreciate the difficulty in determining simple endocrine markers for exercise monitoring. Next, Pierce et al. address the important concept that women and men may have different hormonal responses to similar exercise training. During and after performing a loaded squat protocol, the two sexes demonstrated a similar hierarchy of serum proteins in the 3 GH molecular weight (MW) fractions (>60; 30-60; <30 kDa), but distinct response kinetics. Additionally, women and men differed in the IGF-1 MW fraction, and therefore specific IGF-1 superfamily members, that increased with the exercise intervention. In Nindl et al., some of these same authors compared concentration changes of blood and muscle interstitial IGF-1 and IGF binding proteins (IGFBPs) (collected *via* microdialysis probes in the vastus lateralis) pre- and post-unilateral jumping until exhaustion on a sledge. This study was only performed in men, but it demonstrated notable differences in local versus systemic IGF-1 and IGFBP responses to exercise. Combining these concepts of studying women and men and assessing local and systemic hormonal effects of exercise interventions is important for future study design to better inform effective, sex-specific training protocols.

In 2014, the International Olympic Committee (IOC) coined the term, “Relative Energy Deficiency in Sport” (RED-S), to highlight the myriad health and performance consequences of low EA in both female and male athletes. The IOC authors acknowledged that menstrual dysfunction and bone decrements are well-known sequelae of low EA (i.e., Female Athlete Triad), but encouraged further research into the other bodily systems and sports performance realms affected by low EA in broader athlete populations (1). In this edition, three papers address this topic. Hooper et al. assessed potential RED-S effects in 7 collegiate female cross-country athletes over 6 months. The women had no significant changes in body composition from pre- to post-Fall cross-country season, but had decreases in 25OH vitamin D and ferritin. Prior to outdoor Track season, a time of recovery, body mass increased, along with resting metabolic rate (RMR) and the aforementioned blood markers in the group as a whole. Interestingly, significant triiodothyronine (T3; a marker of low EA) changes were not observed. Multiple athletes reportedly had somewhat low EA, but only one experienced severely decreased RMR and performance, which improved with a nutritional and training intervention. Her ferritin was elevated, possibly indicating an inflammatory response (2). Stenqvist et al. studied similar markers during 4-weeks of intense training in 20 adult male cyclists. Short-term additions of high intensity training sessions to their baseline training over a month led to increased endurance performance and total testosterone. T3 and RMR decreased, and cortisol increased, as seen in prior work on athletes with RED-S (3). The group as a whole did not significantly change their testosterone:cortisol ratios, but individual ratio increases positively correlated with performance improvement. This is consistent with prior work on overtraining syndrome (OTS) (4). While the former case series and the latter intervention trial are both small, they illustrate the importance of considering multiple markers when assessing RED-S risk and of understanding that athletes have different EA thresholds for optimal functioning.

Hackney addressed RED-S versus OTS in his review on hypogonadism in exercising men, discussing variable causes of hypogonadism including hormonal inhibitory factors of stress, overtraining, inadequate EA, current or historic anabolic androgenic steroid use, traumatic brain injury, and chronic exercise without performance decrements [Exercise Hypogonadal Male Condition (EHMC)]. Distinguishing causes of hypogonadism is critical, as some of them are truly dysfunctional, but EHMC is debatably an appropriate adaptation, as seen among highly physically active men in non-industrial populations (5). Hackney’s emphasis on different causes and consequences of low testosterone in male athletes is important in the RED-S versus OTS debate. Cadegiani and Kater (6) attempted to further clarify markers of training adaptation versus OTS in their analyses of the Endocrine and Metabolic Responses on Overtraining Syndrome (EROS) trial. In EROS, 25 healthy athletes, 14 OTS athletes, and 12 non-athletes (all male) were compared. OTS athletes were selected using interviews, performance metrics, biochemical testing, and other OTS guidelines; 117 markers were assessed in the three groups (7). In the EROS-CORRELATIONS paper (Cadegiani and Kater), the authors assessed the interplay of biomarkers to better differentiate OTS from healthy athletes. Fat mass inversely correlated with testosterone:estradiol ratio, which predicted, together with cortisol, prolactin and GH responses to stimulation, the measured RMR:predicted RMR ratio. Hypothalamic response to stress stimulation was diffuse and not hormone-specific. In the EROS-PREDICTORS paper (Cadegiani and Kater), carbohydrate intake predicted earlier hormonal responses to stimulation. Speed of muscle recovery after training was directly predicted by any source of caloric intake; protein intake predicted improved body composition.

The last two papers introduced new parameters for exploration. Eklund et al. examined the second to fourth digit ratio (2D:4D) – suggested to result from higher prenatal testosterone exposure (8) –in 104 female Olympic athletes and 117 non-athlete controls, along with serum and urine steroid profiles. The right hand 2D:4D was lower in athletes versus controls and associated with better strength and endurance performance. The ratio was negatively correlated with some urine testosterone metabolites but not with serum testosterone levels, possibly from differences in androgen metabolism only detected *via* urinary sampling. While compelling, the proposition that fetal androgen exposure predicts physical performance should be viewed with caution, as there are numerous contributors to athletic success. This may be an area for further exploration in some sports disciplines. Last, Munoz et al. considered the use of irisin, a muscle-contraction-induced myokine, as a metabolic biomarker of health-related fitness, testing normal- and overweight female students. Hand grip strength and irisin concentration correlated in the overweight group, but further studies are warranted to determine relevance in athletes.

In conclusion, as highlighted in Hackney’s review, early, sensitive, and specific tools are needed to clearly distinguish dysfunction (e.g., OTS or low EA) from adaptation in athletes, accounting for gender and sport disciplines. New potential markers of interest have been presented in this edition, but they require validation with more comprehensive exploration at the local and systemic level in larger cohorts, considering the high inter-individual variation in highly functioning athletes. Studies in diverse populations of athletes and controls should attempt to employ the most standardized methodology for hormonal and metabolic assessment to further unveil adaptations that occur in athletes.

## Author Contributions

All authors contributed equally to this manuscript. All authors contributed to the article and approved the submitted version.

## Conflict of Interest

Author FAC was employed by Applied Biology Inc.

The remaining authors declare that the research was conducted in the absence of any commercial or financial relationships that could be construed as a potential conflict of interest.

## Publisher’s Note

All claims expressed in this article are solely those of the authors and do not necessarily represent those of their affiliated organizations, or those of the publisher, the editors and the reviewers. Any product that may be evaluated in this article, or claim that may be made by its manufacturer, is not guaranteed or endorsed by the publisher.
